# Influence of dual vitamin D therapy on the outcome in patients with moderate-to-severe acute anterior circulation cerebral infarction and vitamin D insufficiency

**DOI:** 10.3389/fnhum.2025.1629366

**Published:** 2025-09-30

**Authors:** Jing Wei, Wenying Cao, Shuchun Huang, Yuxing Feng, Cheng Li

**Affiliations:** Neurology Department, Beibei Hospital of Chongqing Medical University, Chongqing, China

**Keywords:** vitamin D, dual supplementation therapy, vitamin D deficiency, acute cerebralinfarction, prognosis

## Abstract

**Objective:**

To observe whether dual vitamin D supplementation therapy can improve the prognosis of patients with moderate-to-severe anterior circulation acute cerebral infarction accompanied by vitamin D deficiency.

**Methods:**

A retrospective analysis was conducted on 36 patients with moderate-to-severe anterior circulation acute cerebral infarction accompanied by vitamin D deficiency. They were divided into a dual vitamin D supplementation therapy group and a control group. The 90-day mRS scores of the two groups of patients and the occurrence of hypercalcemia were compared.

**Results:**

The difference in the number of mRS 0–3 cases at 90 days was statistically higher in the dual vitamin D supplementation group than in the control group (*P* < 0.05). No hypercalcemia was observed in either group.

**Conclusion:**

Dual vitamin D supplementation improves outcomes in patients with moderate-to-severe anterior circulation acute cerebral infarction accompanied by vitamin D deficiency, and is relatively safe.

## 1 Introduction

Acute cerebral infarction is caused by the sudden interruption of cerebral blood flow due to various reasons, resulting in brain tissue necrosis (Feske, 2021). It has a high incidence, mortality, and disability rate. According to the occluded blood vessels, it can be divided into anterior circulation cerebral infarction caused by internal carotid artery system occlusion and posterior circulation cerebral infarction caused by vertebrobasilar artery system infarction (Herpich and Rincon, 2020). According to the degree of neurological deficits, it can be classified into mild, moderate, and severe cerebral infarction. Generally, patients with a National Institute of Health stroke scale (NIHSS) greater than 4 are defined as moderate-to-severe cerebral infarction (Naess et al., 2016). Currently, the early treatment of cerebral infarction includes intravenous thrombolysis and endovascular treatment, but the treatment time window is limited (Diener et al., 2013; Yuan et al., 2014). The main treatment methods for most patients are antithrombotic therapy and lipid-regulating and plaque-stabilizing therapy. Some studies have also pointed out that certain neuroprotective drugs have a certain effect. Vitamin D is a new type of immunomodulatory element and is currently proven to be related to many neurological diseases (Alfieri et al., 2017). It can exert its effects through anti-inflammation, anti-oxidation, regulation of neurotransmitters, and regulation of calcium homeostasis. This study aims to observe the effectiveness of dual vitamin D supplementation therapy on the prognosis of patients with moderate-to-severe anterior circulation acute cerebral infarction accompanied by vitamin D deficiency, in order to provide more treatment options for cerebral infarction patients.

## 2 Data and methods

### 2.1 General data

A total of 36 patients with moderate-to-severe anterior circulation acute cerebral infarction who were admitted to the Ninth People’s Hospital of Chongqing from March 2019 to August 2022 within 24 h of onset were included as the research subjects. The inclusion criteria were as follows: (1) Confirmed as anterior circulation acute cerebral infarction by cranial CT or MRI. (2) National Institute of Health stroke scale (NIHSS) ≥4 points at admission. (3) Serum vitamin D <30 ng/ml at admission. (4) emergency endovascular treatment were not performed. (5) Modified Rankin Scale (mRS) with the score of 0–1 before the onset. A retrospective analysis was performed on the included subjects. Relevant treatments such as past medical history, electrocardiogram, blood routine, coagulation function, serum vitamin D, biochemistry, and neurological function evaluated by National Institute of Health stroke scale (NIHSS) were collected. According to different treatment methods, they were divided into the dual vitamin D supplementation group and the control group.

### 2.2 Treatment methods

All patients included in the study were given treatments such as intravenous thrombolysis, anti-platelet aggregation, and lipid-regulating and plaque-stabilizing therapy according to the treatment guidelines for acute cerebral infarction based on their conditions (Hilkens et al., 2024). The treatment group received dual vitamin D supplementation therapy. The dual vitamin D supplementation regimen was as follows: (1) One-time intramuscular injection of vitamin D2 (for 20 ng/ml ≤ blood vitamin D <30 ng/ml, 200,000 units of vitamin D2 were given; for blood vitamin D <20 ng/ml, 400,000 units of vitamin D2 were given). (2) Calcitriol 0.5 μg was given orally or by nasogastric tube once a day. The control group was given calcitriol 0.5μg orally or by nasogastric tube once a day.

### 2.3 Observation indicators

The 90-day modified Rankin Scale (mRS) scores of the dual vitamin D supplementation group and the control group were observed. Modified Rankin Scale (mRS) scores of 0–3 points (mild to moderate disability) were defined as a good prognosis, and modified Rankin Scale (mRS) scores of 4–6 points (severe disability or death) were defined as a poor prognosis. The study compares the difference in good prognosis and occurrence of hypercalcemia between different vitamin supplement treatment groups. The differences in age, gender, pre-onset mRS, NIHSS at admission, serum vitamin D, blood calcium, D-dimer, fibrinogen, white blood cells, platelets, hemoglobin, creatinine, uric acid, and GFR between the two groups were compared. The differences in combined hypertension, type 2 diabetes, and hyperlipidemia between the two groups were also compared (Table 1).

**TABLE 1:** Comparison of data from different treatment groups.

Variables	Treatment group	Control group	*P*
Age (years old)	78.4 ± 9.7	75.8 ± 11.0	0.457
Male (*n*, %)	7 (38.9)	7 (38.9)	1.000
Intravenous thrombolysis (*n*, %)	4 (22.2)	11 (61.1)	0.018
NIHSS at admission (score)	17.8 ± 6.5	17.7 ± 6.3	0.959
Type 2 diabetes (*n*, %)	2 (11.1)	3 (16.7)	0.63
Hypertension (*n*, %)	11 (61.1)	13 (72.2)	0.48
Hyperlipidemia (*n*, %)	5 (27.8)	3 (16.7)	0.423
Smoking (*n*, %)	2 (11.1)	1 (5.6)	0.546
Alcohol abuse (*n*, %)	0 (0)	0 (0)	1.000
Atrial fibrillation (*n*, %)	13 (72.2)	12 (66.7)	0.717
Serum Vitamin D on Admission (ng/ml)	15.4 ± 6.9	13.4 ± 4.6	0.313
Blood calcium on admission (mmol/L)	2.11 ± 0.12	2.18 ± 0.14	0.125
hypercalcemia cases (*n*, %)	0 (0)	0 (0)	1.000
D-dimer (mg/L)	2.30 ± 2.44	2.30 ± 2.65	0.998
Fibrinogen (g/L)	3.28 ± 1.33	3.00 ± 0.98	0.482
Leukocytes (×10^9^/L)	8.25 ± 3.14	7.87 ± 2.71	0.701
Platelets (×10^9^/L)	195.8 ± 73.2	210.2 ± 78.1	0.574
Hemoglobin (g/L)	125.6 ± 16.9	126.4 ± 13.2	0.87
Creatinine (umol/L)	93.2 ± 35.9	79.4 ± 33.7	0.243
Uric Acid (umol/L)	388.6 ± 108.3	363.2 ± 134.1	0.537
GFR (ml/min)	64.8 ± 19.9	73.0 ± 34.8	0.39
Premorbid mRS = 0 (*n*, %)	14 (77.8)	15 (83.3)	0.674
90 days mRS of 0–3 (*n*, %)	6 (33.3)	1 (5.6)	0.035
90 day mortality (*n*, %)	4 (22.2)	4 (22.2)	1

### 2.4 Statistical methods

SPSS 26.0 statistical software was used for statistical analysis of the data. Continuous variables are expressed as mean ± SD (x ± s) with *t* test, categorical variables are expressed as rate (%) with χ^2^ test. *P* < 0.05 indicated that the difference was statistically significant. Given the limited sample size and the exploratory nature of this retrospective study, no adjustments for multiple comparisons were applied. Similarly, multivariate adjustments for baseline covariates were not performed due to the risk of overfitting and the reduced reliability of such models in small samples.

## 3 Results

The number of cases with 90-day mRS of 0–3 was higher in the dual vitamin D supplementation group than in the control group (6 [33.3%] vs. 1 [5.6%]; *P* < 0.05). The absolute risk difference was 27.8% (95% CI: 7.7–47.9%), and the odds ratio was 8.5 (95% CI: 0.94–76.7). The proportion of patients who received thrombolysis was significantly higher in the control group than in the treatment group (*P* = 0.018). No hypercalcemic cases occurred in either group. There was no statistical difference in age, gender, premorbid mRS, National Institute of Health stroke scale (NIHSS) at dmission, serum vitamin D, blood calcium, D dimer, fibrinogen, leukocyte, platelets, hemoglobin, creatinine, uric acid, GFR between the two groups (*P* > 0.05). There was no statistical difference between the two combinations with hypertension, type 2 diabetes and hyperlipidemia (*P* > 0.05).

## 4 Discussion

Vitamin D is a fat-soluble vitamin that functions as a steroid hormone. The inactive vitamin D precursor first undergoes 25-hydroxylation in the liver to form 25-hydroxyvitamin D [25(OH)D], which is the main circulating form of vitamin D (Ellison and Moran, 2021). 25(OH)D is further hydroxylated by 1-α-hydroxylase in the kidneys to produce 1,25 - dihydroxyvitamin D [1,25(OH)_2_D], which is the active form of vitamin D. The main function of vitamin D is to maintain calcium and phosphorus metabolism (Buell and Dawson-Hughes, 2008; Garcion et al., 2002). However, vitamin D receptors (VDR) have been found in many target cells, including endothelial cells, vascular smooth muscle cells, and most immune cells. Vitamin D receptors (VDR) is also widely distributed in neurons and glial cells (Garcion et al., 2002). Therefore, vitamin D is involved in many brain metabolic processes, including neuro-immune regulation, regulation of neurotrophic factors, neuroprotection, nerve repair, and brain development (Buell and Dawson-Hughes, 2008; Fernandes de Abreu et al., 2009). Vitamin D participates in neuroprotection through multiple pathways, including antioxidant mechanisms, regulation of immune responses, regulation of calcium homeostasis, inhibition of pro-inflammatory agents, and detoxification (Brouwer-Brolsma and de Groot, 2015; Gezen-Ak et al., 2014; Landel et al., 2016). Early studies on vitamin D signaling in the brain have demonstrated its regulatory effect on the levels of neurotrophic factors such as nerve growth factor (NGF), glial cell -derived neurotrophic factor (GDNF), neurotrophin 3 (NT3), and neurotrophin 4 (NT4) (Aspell et al., 2018). Active vitamin D inhibits the expression of inducible nitric oxide synthase in the spinal cord and brain, which promotes the production of nitric oxide (Garcion et al., 1997). High nitric oxide levels are considered to be involved in inflammatory diseases (Zhang et al., 2012), and the inflammatory response is an important pathophysiological process of nerve injury after stroke. At the same time, vitamin D can inhibit the production of inflammatory factors such as interleukin 6 (IL-6) and tumor necrosis factor α(TNF-α), resulting in an effect on stroke progression (Balden et al., 2012). Vitamin D deficiency (<20 ng/mL) is common in patients with ischemic stroke (Tu et al., 2014). Many domestic and foreign studies have shown that vitamin D deficiency is related to the onset, severity, and outcome of ischemic stroke. These studies have shown that low systemic vitamin D levels are associated with an increased severity of ischemic stroke at admission (Aubail et al., 2013; Wang et al., 2014), a poor functional prognosis at discharge, a higher risk of depression at 1 month (Alfieri et al., 2017; Han et al., 2015), a higher incidence of cognitive impairment (Chen et al., 2018; Yalbuzdag et al., 2015), and a higher risk of death at 1 year (Daubail et al., 2014).

Previous studies have shown that vitamin D supplementation has a neuroprotective effect. Atif et al. (2013) conducted relevant experiments in a primary culture of cortical neurons model with oxygen-glucose deprivation (OGD) and a transient middle cerebral artery occlusion (MCAO) rat model. The OGD model experiment showed that vitamin D could significantly reduce neuron death. The transient MCAO model experiment showed that vitamin D combined with progesterone could reduce the infarct volume and improve neurological function. Gupta A et al. conducted a randomized controlled open-label trial (Gupta et al., 2016). A total of 53 patients with acute ischemic stroke with baseline 25(OH)D <30 ng/ml were randomly divided into two groups. One group received vitamin D and calcium supplementation (*n* = 25), and the other group was the control group (*n* = 28). The proportion of patients with a good prognosis in the intervention group was higher (OR = 1.9, 95%CI = 0.6–6.4, *P* = 0.31). Compared with the control group, the survival probability of the intervention group was higher (83.8%, CI = 62.4–93.6 vs. 59.5%, CI = 38.8–75.2. *P* = 0.049). The adjusted hazard ratio (HR) was 0.26 (95%CI = 0.08–0.9, *P* = 0.03), indicating that vitamin D and calcium supplementation may have potential benefits for the outcome of ischemic stroke. However, some studies have also shown negative results. In 2012, an animal experiment was reported (Balden et al., 2012). Adult female rats were fed a vitamin D-deficient (VDD) diet for 8 weeks and then underwent middle cerebral artery occlusion. It was shown that the cortical and striatal infarct volumes of the animals fed the vitamin D-deficient (VDD) diet were significantly increased. The sensorimotor behavior test showed that the post-stroke behavioral disorders of the vitamin D-deficient (VDD) animals were more severe than those of the control group. Vitamin D was injected for supplementation at 4 h after stroke and every 24 h thereafter, but vitamin D supplementation treatment did not improve the infarct volume and behavioral ability of the experimental rats.

In this study, the number of cases with 90 days mRS of 0–3 (mild to moderate disability) was higher in the treatment group than in the control group [8 (44.4%) vs. 2 (11.1%), *P* < 0.05], with a statistically significant difference, suggesting that dual vitamin D supplementation therapy can improve the prognosis of patients with moderate-to-severe anterior circulation acute cerebral infarction accompanied by vitamin D deficiency. The thrombolysis rate was higher in the control group than in the dual vitamin D supplementation group (61.1% vs. 22.2%, *P* < 0.05), a difference potentially attributable to the limited sample size. Despite this potential confounding factor, the control group nevertheless exhibited worse outcomes than the dual supplementation group. This finding further supports the efficacy of dual vitamin D supplementation therapy. There were no adverse reactions such as hypercalcemia and severe arrhythmia in both groups of patients, indicating that dual vitamin D supplementation therapy has high safety.

Building upon the established neuroprotective properties of vitamin D supplementation, the present findings are largely consistent with prior research. This study reinforces the favorable association between vitamin D supplementation and improved functional outcomes in stroke patients with vitamin D deficiency, and further demonstrates the superiority of a dual vitamin D supplementation regimen over oral supplementation alone. A novel contribution of this work is the identification of a correlation between dual vitamin D supplementation and improved stroke prognosis, suggesting that this combinatorial interventional strategy warrants further scientific attention. Currently, there is no consensus on optimal vitamin D supplementation protocols, with ongoing debate regarding dosage, formulation, and administration routes (Zemp et al., 2025). In contrast to the predominant focus on oral supplementation in the literature, the present study introduces an intramuscular vitamin D_2_ injection component, which may offer new insights for achieving rapid and sustained neuroprotective effects. Several limitations should be noted, including the retrospective design, modest sample size, and absence of standardized treatment protocols, all of which may increase susceptibility to confounding factors. Future prospective studies with larger sample sizes are needed to validate these observations and elucidate the underlying neurobiological mechanisms.

## 5 Conclusion

The efficacy of vitamin D supplementation in improving prognosis after cerebral infarction remains inconsistent across previous studies, which may be attributed to variations in supplementation regimens–such as the use of vitamin D_2_, vitamin D_3_, or alfacalcidol alone. In the present study, combined supplementation with vitamin D_2_ and calcitriol was associated with a higher rate of favorable outcomes. This suggests that simultaneous sufficiency of both 25(OH)D and 1,25(OH)_2_D may be necessary to facilitate the timely activation of vitamin D-mediated neuroprotective and neural repair mechanisms. Overall, this study introduces an innovative dual vitamin D supplementation and provides novel insights into the role of vitamin D in cerebral infarction. Nonetheless, as a retrospective study with a small sample size, this research has inherent limitations. The therapeutic efficacy and underlying mechanisms of this combined regimen in acute anterior circulation stroke require validation through larger, randomized clinical trials.

## Data Availability

The original contributions presented in this study are included in this article/supplementary material, further inquiries can be directed to the corresponding author.
